# Saline Transfusion, Hemorrhage and Shock

**Published:** 1901-04

**Authors:** J. L. Campbell

**Affiliations:** Atlanta, Ga.


### SALINE TRANSFUSION, HEMORRHAGE AND SHOCK.

BY J. L. CAMPBELL, M.D.,
ATLANTA, GA.

Saline transfusion may be performed in three ways: First, by direct transfusion into superficial vein; second, by hypodermatoclysis or subcutaneous transfusion, and third, by enteroclysis, rectal injections or enemas.

We derive the greatest benefit from saline transfusion in the treatment of hemorrhage, shock and collapse, but it is by no means limited to these conditions. It is of great value in any case where there is absorption of ptomains, uremia and malarial toxemia.

In the treatment of hemorrhage and collapse, the action is for the most part mechanical, filling up the depleted vessels, thus

* Read before the Atlanta Society of Medicine, March 7, 1901.

giving volume to the blood, preventing flabbiness of the muscles, and especially of the heart. The deficiency of the corpuscles will soon be restored unless some serious disease exists.

In shock, the influence is remarkable. Patients react more readily than to almost any other remedy. In the collapse of Asiatic cholera, cholera infantum, or any disease where the fluids of the body have been evacuated, reducing the volume and increasing the density of the blood, saline transfusion act in two ways: first, restores the wasted blood plasms, thus increasing the volume of the blood, restores the arterial tension and the action of the excreting organs; second, it dilutes the ptomains that are absorbed from the intestines and other sources, so that they can be more easily excreted. The action is practically the same when used in the treatment of the septicemias, as here the poison is diluted and glandular function restored, eliminating the poison more rapidly. Care must be taken, however, not to give it where there is too great arterial tension, as convulsion may be produced, due, I suppose to the overfilling of the vessels and cerebral pressure. The operation is quite simple and can be done almost anywhere. We have repeatedly done it here in negro cabins about the city with the very best results.

The technique must be most carefully observed, as only a slight break may result fatally to the patient. First, the solution must be thoroughly prepared. Use 0.6 per cent. solution of sodium chloride, made by adding ʒi of salt to the quart of water. The water must be boiled, the salt added and then strained. When operating where the best of aseptic precautions cannot be observed, I prefer to bring the solution to a boil after adding the salt, and strain a second time. The vessels and towels must be thoroughly sterilized. Ludwig suggests that three or five per cent. of sugar may be added for the nutritive effect, but we have not yet tried it. After the solution has been thus prepared and set aside in a sterilized vessel to cool, the apparatus and instruments should be made ready. These should be sterilized in a 0.6 per cent. solution of sodium carb.

It is best not to use carbolic acid for the instruments as it might be gotten into the saline solution. While the instruments are being sterilized select the vein most suited for the occasion—any superficial vein will do, but the median basilic is most accessible. After selecting the vein, prepare the place as for any other operation—wash well with soap and water, then with 1–1000 bichloride, and finally with alcohol or ether. The vein is exposed by making an incision along its course, varying in length, according to the thickness of the cellular tissue. Expose about three-fourths of an inch of the vein, then pass a ligature, preferably thin catgut, around the vein at the proximal and distal end of the incision. Let them remain untied until the solution is at the proper temperature 42° C. or 105° or 106° F. Then tie the distal ligature and open the vein on the handle of a scalpel or tenaculum, and introduce the canula (which will be described later), and tie it in with a thin silk ligature. The canula will generally fill with blood, if not the amount of air introduced will not be sufficient to do harm. An assistant must be present with a syringe ready to begin work, as delay might admit too much air, or cause slight clotting of the blood, which would prove fatal.

The introduction of air is to be avoided as far as possible, but I have seen a considerable quantity introduced without harm, and have put as much as three or four cubic inches into a dog's veins without injury. There is always a considerable number of bacteria in the air, and for that, if for no other reason, it should be excluded. Use enough fluid to get the result required without regard to quantity. So far we have only mentioned the intravenous transfusion, but where the case is not too urgent, as a makeshift, where the vein cannot be found, or as a means to prevent shock in long-continued operations, where it can be used during the operation, the subcutanous method is advisable. Some authorities prefer this method altogether, but it is more reasonable to believe that where the fluid is put directly into the circulation, the effect will be much more prompt than where the vessels are compelled to take it up from the tissue. One advantage, however, in the subcutanous method is, that brandy can be added, thus securing stimulation.

Saline enemas should never be neglected and should be used where the indications are not sufficiently urgent for the other means, and in conjunction with the others. In these enemas one or two cups of strong coffee may be used with great benefit. This preparation must be as hot as can be well borne by the patient.

Intra-peritoneal injections are recommended, but I have never seen it tried. In an article some time since, in the *London Lancet*, an operator recommended filling the peritoneal cavity with saline solution after a section, and reported considerable success.

The apparatus used by us in doing this operation is quite simple. However, the apparatus does not make much difference, as a fountain or Davidson's syringe can just as well be used.

The instrument I prefer is simply an S-shaped probe-pointed canula, with a slight shoulder near the end to prevent its slipping from the vein. This fits over a nozzle with a faucet, which in turn fits into a rubber tube about 8 or 10 in. long, and this is connected by a glass tube to a second piece of the same length. This glass joint allows us to detect any air that might be about to enter the veins. Altogether the tube is 18 or 20 in. long. To force the fluid into the vein, use a glass syringe with a nozzle slightly bulging, so as to fit snugly and to be slipped in and out of the tube easily. This syringe holds four or five ounces of fluid, is filled and connected with the rubber tube, the air expelled, the canula having been put in the vein as described above, tube connected with the canula and the fluid gently forced into the circulation. When the syringe is empty, remove the tube from the nozzle with the thumb and finger, holding it tightly to prevent the entrance of air, or escape of blood. As assistant fills the syringe from the vessel near by, which stands in a pail of hot water to keep up the proper temperature. The syringe is again connected and a few vigorous knocks with the knuckles on it will cause any air that might have entered to rise to the top of the syringe.

When one is already prepared, with everything previously sterilized, the operation can be done in a very short time, and the results are so gratifying that if only tried it will not be abandoned.

For the subcutaneous method use a sharp aspirating needle connected with the same apparatus as for intravenous transfusion, or with a fountain syringe. Prepare the surface as above described (soap and water, bichloride and alcohol), then pinch up as large a fold of skin as possible, and plunge the needle in so that it will rest in the subcutaneous tissues. It is not necessary to use cocain, as the operation is not painful. Allow the fluid to flow slowly, as too strong a pressure will tear the tissue. Quite a large tumor will at first form, but will soon disappear if the fluid is absorbed. Any point will do except about the neck, as the patient may choke from edema, which might follow the operation. The situation best suited is the breast, flank or inside of the thigh. Abscesses have rarely followed except where the operator was neglectful of his technique. The same care is to be exercised as with intravenous transfusion.

Rectal transfusion can be used without the precautions necessary with the other methods. They are rarely evacuated, and can nearly always be retained if a hypodermic of morphine is given at the same time. Use a fountain syringe, and if possible a soft rubber catheter so as to put the solution as high up in the rectum as possible: allow the water to flow in slowly, and use one or two quarts. It is readily absorbed.

---

KING EDWARD VII. has appointed Lord Lister to be Sergeant-Surgeon in Ordinary and Sir William MacCormac and Sir Thomas Smith to be Honorary-Sergeant-Surgeons. Honorary Physicians to the King: Dugal McEwen, M.D.; Sir James Dennet, Inspector-General of Hospitals and Fleets; Sir John Watt Reid, Director-General of the Medical Department of the Navy (retired); Adam B. Messer, M.D., Inspector of Hospitals and Fleets; Henry C. Woods, M.D., Inspector-General of Hospitals and Fleets (extra). Honorary Surgeons to the King: Sir James Jenkins, Inspector-General of Hospitals and Fleets; Timotheus J. Haran, Inspector-General of Hospitals and Fleets; Sir James N. Dick, Director-General of the Medical Department of the Navy; William H. Lloyd, Inspector-General of Hospitals and Fleets; Alfred G. Delmage, Deputy Inspector-General of Hospitals and Fleets.—*Ex.*

